# Calcium Electroporation of Equine Sarcoids

**DOI:** 10.3390/ani10030517

**Published:** 2020-03-19

**Authors:** Stine K. Frandsen, Julie Gehl, Trine Tramm, Martin S. Thoefner

**Affiliations:** 1 Center for Experimental Drug and Gene Electrotransfer (C*EDGE), Department of Clinical Oncology and Palliative Care, Zealand University Hospital, Sygehusvej 10, 4000 Roskilde, Denmark; 2 Department of Clinical Medicine, Faculty of Health and Medical Sciences, University of Copenhagen, Blegdamsvej 3B, 2200 Copenhagen N, Denmark; 3 Department of Pathology, Aarhus University Hospital, Palle Juul-Jensens Boulevard, 8200 Aarhus N, Denmark; 4 Hørsholm Hestepraksis, Kongevejen 124, 3480 Fredensborg, Denmark

**Keywords:** calcium electroporation, sarcoid, equine, horse, response, biopsy

## Abstract

**Simple Summary:**

Sarcoids are skin tumors on horses where better treatment options are needed. Calcium electroporation is a novel anti-cancer treatment where calcium is injected into the tumor, followed by brief electric pulses (electroporation) that transiently open the cell membrane and allow uptake of calcium. This, in turn, induces cell death. In this study, we aimed to investigate the safety and effect of calcium electroporation on sarcoids. Thirty-two sarcoids in eight horses were treated once or twice with calcium electroporation and followed for 12–38 weeks with size-measurement and clinical photographs. The study showed that calcium electroporation is a safe and feasible treatment for sarcoids, including inoperable sarcoids. Six of 27 sarcoids (22%) were completely eliminated, and six further sarcoids (22%) decreased more than 30% in size. No relation between response and location, type, nor size of the sarcoid was observed. Larger studies are needed to further investigate the effect of calcium electroporation on sarcoids.

**Abstract:**

Sarcoids are common equine skin tumors where the risk of recurrence after treatment is high, and better treatment options are warranted. Calcium electroporation is a novel anti-cancer treatment where lethally high calcium concentrations are introduced into the cells by electroporation, a method where short high-voltage pulses induce transient permeabilization of the cell membrane. This study investigated the safety and long-term response of calcium electroporation on sarcoids. Thirty-two sarcoids in eight horses were included. The study suggested that calcium electroporation is a safe and feasible treatment for sarcoids, including inoperable sarcoids. Horses were treated once (2/8) or twice (6/8) under general anesthesia, where sarcoids were injected with 220 mM calcium chloride followed by electroporation with 8 pulses of 100 μs, 1 kV/cm, and 1 Hz. Biopsies were taken prior to treatment. The sarcoid size was monitored for 12–38 weeks after the first treatment. Complete response was observed in 22% (6/27) of treated sarcoids, and partial response in 22% (6/27), giving a 44% total response. Treatment efficacy did not appear to be related to location, type, or size. In all non-biopsied lesions, a complete response was seen (4/4). In conclusion, in this small study, 44% of sarcoids responded with 22% of sarcoids disappearing.

## 1. Introduction

Sarcoids are the most common equine skin tumors. They are benign tumors of mesenchymal origin classified as occult, verrucous, nodular, fibroblastic, or mixed, and, in rare cases, malignant. Sarcoids may develop in any location but are often found in the head and neck area, as well as the ventral abdomen. Sarcoids are normally not lethal by themselves; however, the size and distribution can severely compromise the use and value of the horse and lead to a decision of euthanasia [1,2]. There is no universally effective treatment option for sarcoids where the most frequently used options are conventional excision, laser treatment, cryotherapy, radiotherapy, and chemotherapy, as well as antiviral or immune-modulating treatment. There is a high risk of recurrence; thus, investigations in new therapies are warranted [1,2]. 

Calcium electroporation is a prospective novel anticancer treatment, showing promising response rates in preclinical and clinical trials [3,4,5,6,7]. High calcium concentrations are introduced to the cell cytosol by electroporation, a method where short, high-voltage pulses permeabilize the cell membrane, allowing for passage of non-permeant ions and molecules, e.g., calcium [4,7]. Calcium is a ubiquitous second messenger involved in most cellular processes, including cell death, and, therefore, is tightly regulated by channels, transporters, and intracellular chelators [8]. The high intracellular calcium concentration induced by calcium electroporation causes ATP depletion, mitochondrial dysfunction, and cell death [4,9,10]. Studies in mice have shown cell death through necrosis in several different tumor types [4,5]. Normal cells and tissues have been shown to be less affected by calcium electroporation than cancer cells and tissues [5,11]. Calcium electroporation has also been proven effective in two small human clinical trials on cutaneous metastases, with a complete response rate of 66%, and recurrent head and neck cancer, where one of the six included patients had a complete response [3,6]. Interestingly, a patient with cutaneous metastases from malignant melanoma experienced a systemic immune response with complete remission of treated as well as untreated tumors [12]. Induction of a systemic response has been verified in an in vivo study where mice with a murine colorectal cancer tumor were treated with calcium electroporation and then re-challenged with the same cancer cells and did not develop new tumors [13].

A retrospective study from 2011, investigating the effect of treatment with a chemotherapeutic drug (cisplatin) in combination with electroporation (electrochemotherapy) on equine sarcoids, showed very low recurrence rates (1 of 194 treated sarcoids) [14]. However, due to restrictive regulations on the use of chemotherapeutic drugs in veterinary medicine, the first study investigating calcium electroporation treatment of equine sarcoids was initiated and recently published, showing the method´s safety and short-term effects of the treatment [15]. Sixteen sarcoids in ten horses were treated with calcium electroporation using surface electrodes, followed by surgical removal of sarcoids at 7, 14, and 21 days after treatment. The treatment was well tolerated by all horses. The histological analysis showed necrosis in 13 of 16 sarcoids, and, in nine of these, the fraction of necrosis was higher than 50%. Other main changes in the sarcoids were hemorrhages in 14 of 16 sarcoids, ulceration in 13 of 16 sarcoids, thrombosis in 13 of 16 sarcoids, and calcification in 11 of 16 sarcoids. Surrounding normal tissue seemed less affected by the treatment than the tumor [15].

This study aimed to investigate the safety and long-term effects of calcium electroporation treatment of equine sarcoids. We hypothesized that calcium electroporation would be safe and feasible for the treatment of equine sarcoids, showing a long-term response rate comparable to the ones previously reported.

## 2. Materials and Methods

### 2.1. Study Design

Horses with one or more sarcoids were treated with calcium electroporation to evaluate the long-term effect on the size of the sarcoid. A second treatment could be performed if the sarcoid was not eliminated by the first treatment. The Danish Animal Experiments Inspectorate and The Danish Veterinary and Food Administration (Ministry of Environment and Food of Denmark) were consulted before the study regarding approval. These institutions deemed that since human clinical trials had been completed [3,6], approval was not needed for the current equine study. The study used a similar treatment procedure as in previous human clinical trials [3,6]. All owners received written information about the study and signed informed consent before treatment.

### 2.2. Selection of Animals

Healthy horses with at least one sarcoid able to undergo general anesthesia were included in the study. Rectal temperature, cardiovascular, and respiratory status were examined before general anesthesia, and animals with normal parameters were included in the study. Horses were included in the study prospectively and consecutively. 

### 2.3. Treatment

Horses were treated under general anesthesia. For premedication, horses were sedated with acepromazine 0.03 mg/kg (Plegisil^^®^^), romifidine 0.05 mg/kg (Sedivet^®^), butorphanol 0.015 mg/kg (Torbugesic^®^) i.v., and then ketamine 2 mg/kg (Aniketam^®^) and diazepam 0.05 mg/kg (Stesolid^®^) i.v. for induction of the general anesthesia, and isoflouran 1.8%–2.2% (Attane^®^) for maintenance. The horses were monitored during the procedure.

A highly experienced veterinarian clinically assessed the sarcoids (based on the history of the horse, the appearance, and the location of the sarcoid) before inclusion in the study. All sarcoids were measured and photographed using an iPhone 8 (Apple Inc., Cupertino, CA, USA), hairs around the area were clipped, and standard surgical aseptic preparation was carried out before biopsies were performed (using 8 mm biopsy punch), if assessed reasonable based on size and location of the sarcoid, e.g., if more sarcoids were located close together, not all were biopsied. Sarcoid volume was calculated as ab^2^π/6, where *a* is the largest diameter, and *b* is the largest diameter perpendicular to *a* [3,6]. However, this formula is less appropriate for very thin tumors, so, in cases of thin sarcoids, the volume was calculated as abcπ/6, where *a* is the largest diameter, *b* is the largest diameter perpendicular to *a,* and *c* is the actual thickness of the sarcoid. 

**Figure 1:**
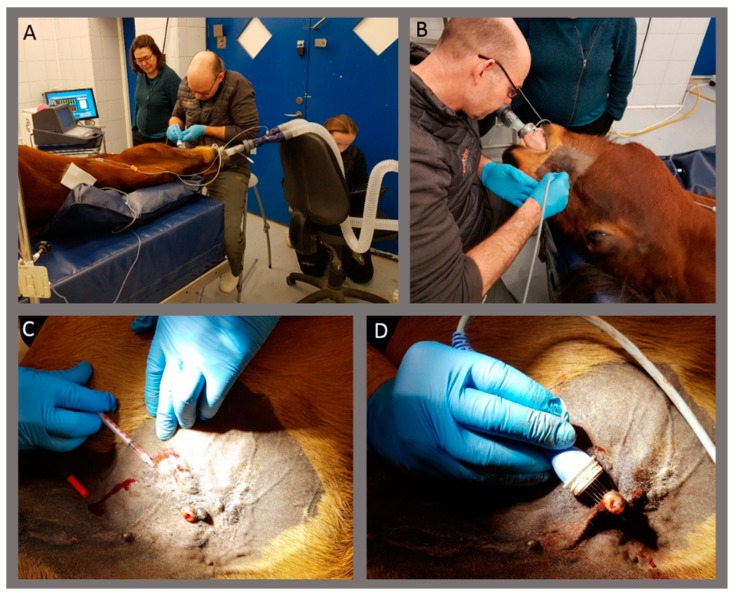
Images of the treatment. Horses were treated in general anesthesia (**A**,**B**). Calcium chloride solution was injected in the sarcoid (**C**), followed by electroporation using needle electrodes (**D**).

Sarcoids were treated by injecting 220 millimolar (mM) calcium chloride solution (500 mM calcium chloride diluted in physiological saline, equals 8.8 g/L calcium chloride) intratumorally, followed by electroporation (Figure 1). Calcium was injected throughout the sarcoid using a 1 mL 29G BD Micro-Fine insulin syringe (Becton Dickinson, Wokingham Berkshire, England) in a volume approximately equivalent to 50% of the sarcoid volume. The goal was to inject calcium in a volume equivalent to 50% of the tumor volume; thus, if calcium solution was spilled during injection (e.g., through the biopsy hole), a similar volume was injected additionally. Immediately after the injection of calcium, the sarcoid was electroporated with 8 pulses of 100 μs, 1000 V/cm, and 1 Hz using a square-wave electroporator (Cliniporator EPS01; IGEA, Italy) and linear needle electrodes (20 mm in length and 4 mm between electrodes; IGEA, Italy). The electrode was moved if needed to electroporate the entire area. In the case of large sarcoids, the area was treated in sections to secure the presence of high calcium concentration in the tissue when electroporating. Horses were treated twice if the sarcoid was not eliminated after the first treatment and if the owners agreed to a second treatment. A second treatment was performed four weeks or more after the first treatment, based on a recommendation from treatments with electroporation in combination with a chemotherapeutic drug (in humans) [16,17] and when feasible for the clinic and owner. Biopsies were only performed before treatment(s) in order to not risk exacerbating tumor growth, which has previously been observed [1]. 

Horses could leave the clinic in their normal habitus a few hours after the procedure, yet some horses stayed at the clinic overnight based on the owner’s decision. 

### 2.4. Follow-up

Sarcoids were measured and photographed after the treatment(s), and the last follow-up, more than 12 weeks after the first treatment (see Table 1), was performed when feasible for the clinic and owner. The percentage change in the largest diameter was determined. Sarcoid areas, deemed as scar tissue at follow-up, were set to a sarcoid diameter of 0 cm, representing a complete response.

### 2.5. Histology

Biopsies were performed before treatment(s), immediately formalin-fixed and paraffin-embedded, with subsequent sections of 3 μm stained with hematoxylin and eosin (HE). A pathologist verified the sarcoid diagnosis and recorded the subtype (verrucous, fibroblastic, etc.) based on morphological appearance, as well as the presence of necrosis, calcification, hyalinization, or ulceration. 

## 3. Results

### 3.1. Included Horses and Sarcoids

**Figure 2:**
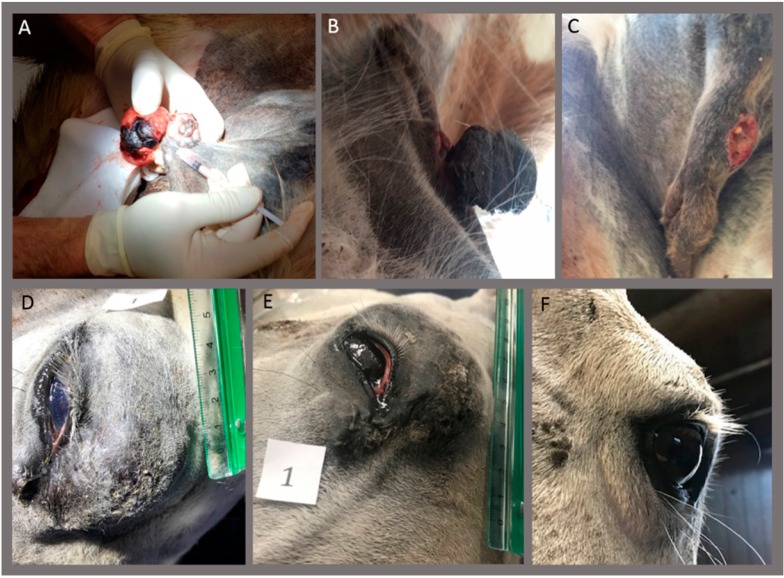
Complete response after calcium electroporation. Horse #4 (**A**–**C**). Pendulating, crusted, non-pigmented sarcoid on the ventral abdomen where the base of the sarcoid was treated once with calcium electroporation (**A**). Four days after the treatment, the sarcoid was starting to fall off (**B**) and completely gone 6 days after treatment (**C**). No regrowth was seen afterward (38 weeks after treatment). A biopsy was performed on the top of the sarcoid, verifying the diagnose. Horse #7 (**D–F**). Sarcoid on the eyelid was treated twice with calcium electroporation with 8 weeks between the treatments. (**D**) before the first treatment and (**E**) before the second treatment. Complete response was observed at follow-up 32 weeks after the first treatment (**F**) - notice the photo is from a caudo-lateral angle. A biopsy was performed before the first and second treatment, with both biopsies verifying the diagnosis.

Thirty-two sarcoids in eight horses (six geldings and two mares), aged 2–15 years, were treated with calcium electroporation, as shown in Table 1. The number of sarcoids varied from 1–9 sarcoids per horse and were situated on the head and neck, ventral abdomen, and extremities, as shown in Table 1 and Table 2. Six horses were treated twice with calcium electroporation with 4–13 weeks between treatments. One horse (#4) was only treated once with calcium electroporation because the sarcoid was completely eliminated after the first treatment (Figure 2A–C). Another horse (#2) was withdrawn from the study after the first treatment and, therefore, only treated once due to multiple large mixed-type sarcoids that were observed when the horse was in general anesthesia and hair clipped. The horse was considered too severely affected and, therefore, withdrawn from the follow-up. 

### 3.2. Safety

Calcium electroporation was well tolerated by all horses. As previously reported for electroporation-based treatments, local muscle contractions occurred when electric pulses were applied [16]. No complications during anesthesia nor calcium electroporation treatment were observed. Two horses that developed a fever for 24–48 h duration and subcutaneous infection received intramuscular penicillin procaine (25,000 IU/kg) and oral meloxicam (0.6 mg/kg) for five days. The remaining horses were clinically unaffected except for local minor to moderate swelling of the treated area. The swelling of the treatment area was observed in all horses after treatment and diminished within a few days. Needle electrodes were used for the electroporation procedure and could be slightly difficult to pull out of the sarcoid due to the firm tissue, pulling the tissue up and letting air enter the biopsy hole.

One of the horses (#1) was euthanized late in the follow-up period due to an accident unrelated to the administered treatment or burden of sarcoids.

### 3.3. Effect

**Figure 3:**
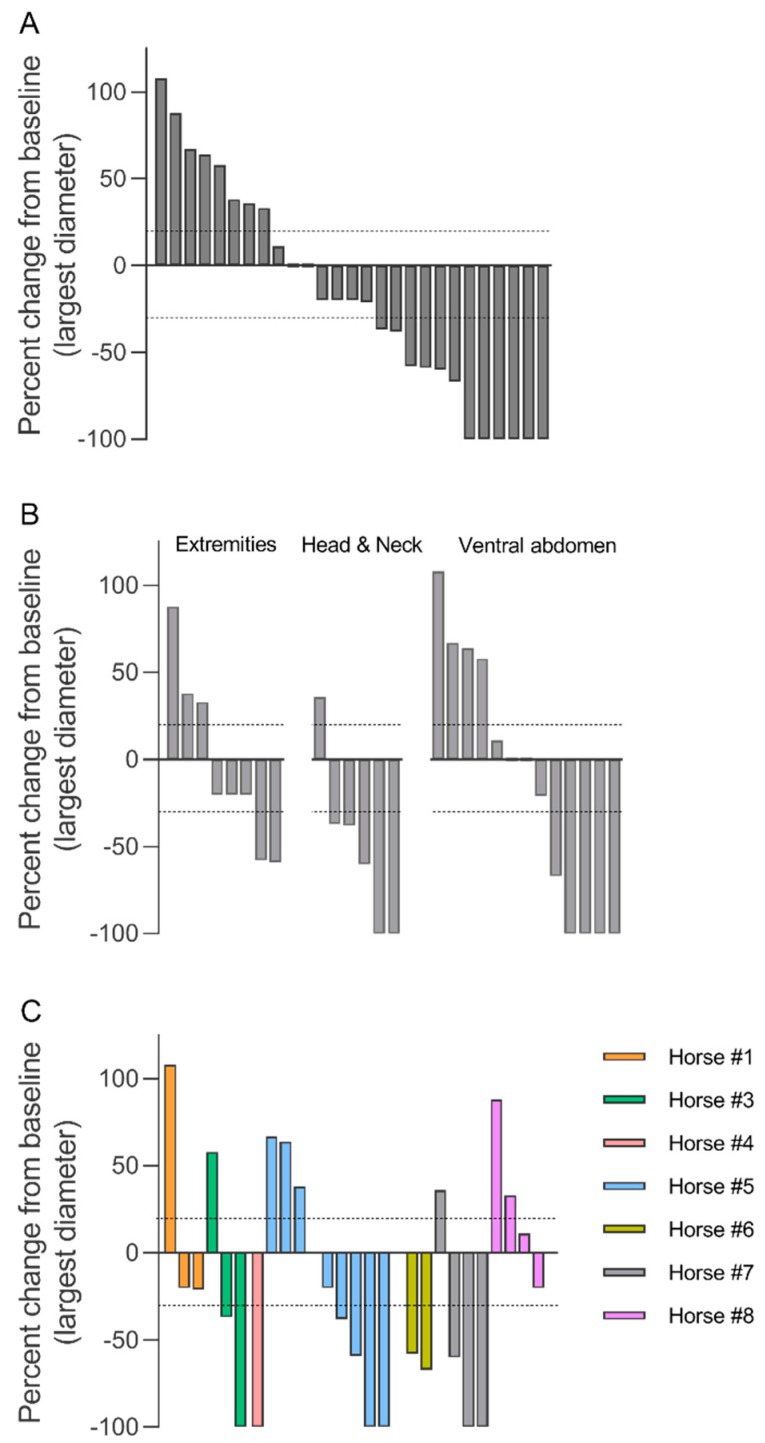
Sarcoid response to calcium electroporation. Percent change in the longest diameter at the last follow-up (12–38 weeks after first treatment). The response of all sarcoids (**A**), showing 8 sarcoids with progressive disease (above 20% increase; dotted line), 7 sarcoids with limited change (up to 30% decrease but not above 20% increase), 6 sarcoids with partial response (more than 30% decrease; dotted line), and 6 sarcoids with complete response (100% decrease). Data is also shown grouped by location of sarcoid (**B**) and individual horse (**C**).

The size of the sarcoids was followed for 12–38 weeks after the first treatment (see Table 1). Figure 3A illustrates the overall sarcoid response by the percentage size change (longest diameter) of the sarcoid from the first treatment to the last follow-up.

Complete response was observed in six of 27 sarcoids (22%) located on the head and neck, as well as the ventral abdomen, on four different horses. In four of these six lesions, no biopsies were performed before any of the two treatments. In one of the sarcoids, which was pendulating with a narrow base, a biopsy was only performed on the top of the sarcoid, while only the base of the sarcoid was treated (Figure 2A–C); thus, no biopsy was performed in the treatment area. Biopsies were performed in all other sarcoids, either before the first or second treatment or before both treatments.

Partial response (more than 30% decrease of the longest diameter) was observed in six of 27 sarcoids (22%) located at the extremities, head and neck, as well as the ventral abdomen, on four different horses. Limited changes in sarcoid size (up to 30% decrease but not above 20% increase) were observed in seven sarcoids (26%) located on the extremities and the ventral abdomen on four different horses. Progressive disease (more than 20% increase) was observed in eight sarcoids (30%) located on the extremities, head and neck, as well as the ventral abdomen, on five different horses. In one of the horses (#5), several new smaller sarcoids were observed on the ventral abdomen at follow-up 26 weeks after the first treatment.

**Figure 4:**
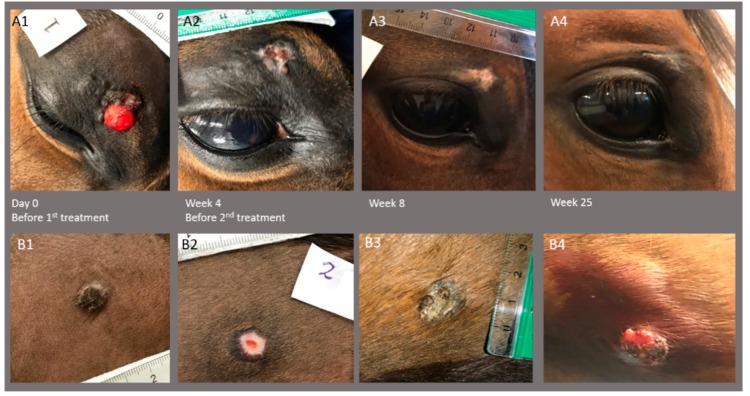
Images of two sarcoids on horse #3 before the first treatment, before second treatment at week 4, and at follow-up 8 and 25 weeks after first treatment. Top row (**A1–4**) shows sarcoid placed on the eyelid with complete response (A4; only scar tissue left), and bottom row (**B1–4**) shows sarcoid placed on the ventral abdomen with the growth of the sarcoid 25 weeks after the first treatment (**B4**). Note that biopsy of the ventral abdomen sarcoid was performed before images were taken at week 4 (**B2**).

**Figure 5:**
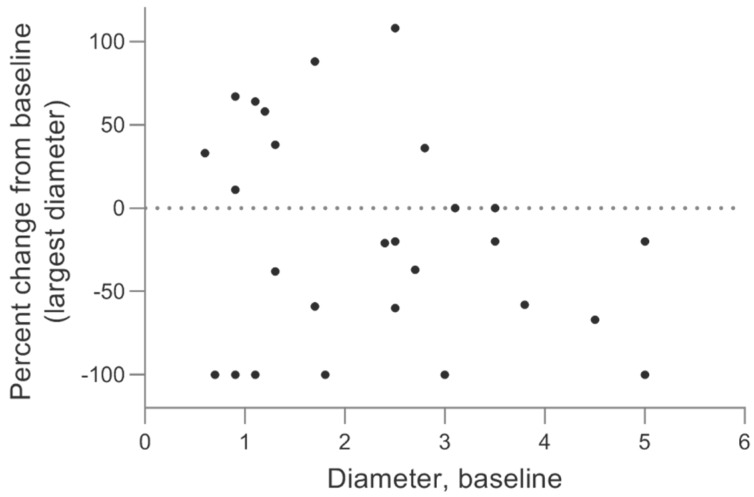
The sarcoid response related to sarcoid size. Percent change of sarcoid size (at the last follow-up) after calcium electroporation treatment(s) on the *y*-axis related to the size of the sarcoid before treatment on the *x*-axis. There does not seem to be a relation between sarcoid size and the likelihood of treatment success with dots evenly spread across the graph.

The response of the sarcoids did not seem related to the location of the sarcoid, as depicted in Figure 3B. The sarcoid response did not depend on the horse as all horses with more than one sarcoid experienced response of some of the treated sarcoids and progression of other sarcoids (Figure 3C and Figure 4). No relation between response and sarcoid size before treatment was observed (Figure 5).

### 3.4. Histology

**Figure 6:**
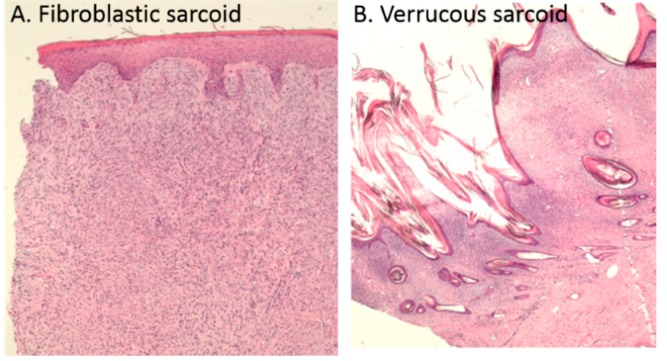
Histological analysis to verify the diagnosis. Light microscope images of H&E sections of fibroblastic sarcoid (40X magnification) (**A**) and verrucous sarcoid (20X magnification) (**B**).

Of the 27 sarcoids (32 treated sarcoids, excluding the five sarcoids treated on the horse that was withdrawn from the study due to multiple large, mixed type sarcoids), 21 sarcoids were biopsied before the first treatment. Of the six lesions not biopsied before the first treatment, two were biopsied before the second treatment; thus, only four lesions were not biopsied. Histological analyses confirmed the sarcoid diagnosis in all biopsied lesions. Analysis of the 21 sarcoids performed before the first treatment showed that 11 (52%) were fibroblastic, and 10 (48%) were verrucous (Figure 6). In general, few changes were observed in the biopsies likely due to the relatively long time span between the biopsies performed (time between the two treatments, ≥4 weeks). Necrosis was only observed in one biopsy, and this was before the first treatment (70% necrosis). Ulceration was observed in five sarcoids before the first treatment, and, of these, four also had ulceration before the second treatment. In the other seven sarcoids, ulceration was observed before the second treatment. A small degree of hyalinization was observed in a few sarcoids (seven before the first treatment, and two before second treatment), and calcification was observed in five sarcoids after the first treatment (in biopsies performed immediately before the second treatment). In two sarcoids on horse #5, hyperkeratosis and pseudo-carcinomatous epithelial hyperplasia, respectively, were observed. Only limited changes in sarcoid size were shown in these sarcoids. No dependency between sarcoid response and type, ulceration, hyalinization, nor calcification was observed.

## 4. Discussion

Sarcoids are common equine skin tumors with many treatment options but no consistently effective treatments. Thus, viable treatment options, where practical and financial aspects are taken into account, are needed. Calcium electroporation is a simple and inexpensive local tumor treatment, proven safe and efficient in human clinical trials on patients with cutaneous metastases from breast cancer and malignant melanoma [3,18], as well as on the recurrent and mucosal head and neck cancer [6]. In a recent study, calcium electroporation was tested on equine sarcoids, where short-term effects of the treatment were observed with calcium electroporation performed preoperatively [15]. In this study, we investigated the safety as well as the long-term effects of calcium electroporation treatment of sarcoids.

Like the clinical trials on humans and the recent equine study, we observed that calcium electroporation is safe and feasible for local treatment of equine sarcoids. Overall, 44% (12/27) of the treated sarcoids responded to the treatment, with more than a 30% decrease in the longest diameter. In the study, investigating short-term effects, nine of 16 sarcoids (56%) treated with calcium electroporation showed more than 50% necrosis 7–21 days after treatment [15]. This was slightly higher than the long-term response we observed, which was likely due to regrowth in some of the sarcoids over time. Sarcoid location, sarcoid type (fibroblastic or verrucous), and sarcoid size before treatment seemed unrelated to treatment response; however, further studies are needed. The correlation between short-term response and sarcoid size before treatment has been previously observed [15].

None of the horses showed a response in all treated sarcoids (if more than one sarcoid was treated), indicating that the response was based on individual parameters of each sarcoid and not host-related factors. Furthermore, this indicated that no systemic immune response was induced, previously seen in a patient with cutaneous metastases from malignant melanoma treated with calcium electroporation [12], as well as a mouse study investigating systemic immune response after calcium electroporation [13]. Further investigations of the induction of a systemic immune response using calcium electroporation would be of great interest for treatments in the human clinical setting, as well as the veterinary setting.

Complete response was observed in 22% (6/27) of the treated sarcoids, which was lower than after surgical removal, where recurrence was reported in 50%–65% of sarcoids [19]. Interestingly, in four of the six lesions with complete response, no biopsies had been performed in the treatment area. Leakage of calcium through the biopsy hole might have reduced the efficacy of the treatment despite the injection of additional calcium. Furthermore, biopsy-induced trauma or irritation is known to exacerbate the sarcoid and induce growth [20], which might also reduce the efficacy of calcium electroporation, although the treatment itself induces some trauma (injection and needle electrodes). However, better response rates were observed in non-biopsied lesions, indicating that trauma induced by the treatment itself did not exacerbate the sarcoids. It would be very interesting to investigate the response rate of calcium electroporation on sarcoids without concurrent biopsies in future studies.

### Limitations

This study showed that calcium electroporation of sarcoids is feasible; however, it can be difficult to disperse calcium in the tough tissue. We, therefore, chose to use insulin syringes to ensure that the needle and syringe stayed together under high injection pressure. Due to the tough tissue, which might result in limited diffusion, we injected calcium in a grid pattern all over the sarcoid to ensure the presence of calcium throughout the sarcoid tissue. Despite the firmness of the tissue, we did not experience any difficulties placing the needle electrode in the tissue, nor the needles becoming blunt after use on several sarcoids (on the same horse). However, sometimes, we had trouble with the generator turning off due to the current in the tissue being too high (above 13 A; note the generator used was an older model). This might happen in some tissue types, but generators that generate higher currents are available, which could potentially solve this problem. In this study, we used needle electrodes, which, sometimes, were slightly difficult to remove, elevating the tissue, and letting air enter through the biopsy hole. This could easily be avoided by pushing the tissue down while pulling the electrode out. Other electrodes could also be used for the treatment. In the previous sarcoid study, surface electrodes were used, which treated 2–3 mm of the sarcoid at each treatment, whereas needle electrodes treat a deeper area of the sarcoid. The effect of the treatments using different electrodes cannot be compared since the endpoints in the two studies are different, but it is important to note the different technical solutions being employed.

Previous treatments of the sarcoids, in this study, were not registered. This might affect the response rate of the sarcoid since it has been shown that recurrence following treatment of sarcoids is associated with exacerbation [20]. Thus, response rates might be higher in previously untreated sarcoids. Registration of previous treatments might be interesting for future studies with calcium electroporation of sarcoids. In this study, we chose also to include four lesions that were not biopsy-verified, although complete certain identification of sarcoids requires histopathological analysis. However, given the experience of the veterinarian, and the fact that all biopsied lesions were histologically verified as sarcoids, we found it credible that the clinical diagnosis of sarcoid of these four lesions was also correct.

The evaluation of a possible new treatment for sarcoids is, of course, based on the response rate, as well as the cost of treatment. Calcium electroporation on horses is performed in general anesthesia and requires electroporation equipment, generator, and electrodes. Although electroporation equipment for veterinary use is reasonably priced, the cost of the treatment is higher than surgical removal. Furthermore, calcium electroporation may not be the first treatment choice for sarcoids based on these few cases since the response rate is slightly lower than after surgical removal. The response rate, in this study, was also lower than the response shown in a retrospective study of electrochemotherapy on equine sarcoids [14]; however, the use of chemotherapeutic agents requires higher safety standards and expensive handling of biological hazard waste. A great advantage of calcium electroporation treatment is, nevertheless, that it is possible in areas where surgical removal is challenging, such as sarcoids placed on the eyelid. In this study, sarcoids located around the eye on horse #7 that could not be surgically removed without removing the eye as well were treated with calcium electroporation with complete response. Thus, calcium electroporation may be a suitable treatment option for inoperable sarcoids.

## 5. Conclusions

This study confirmed that calcium electroporation is safe and feasible for the treatment of equine sarcoids, including inoperable sarcoids. The study is the first to investigate the long-term response rate of calcium electroporation treatment of sarcoids, and it showed 22% (6/27) complete response and 22% (6/27) partial response (defined as more than 30% decrease of the longest diameter). The response of the sarcoids was not related to location, sarcoid type, nor size. Non-biopsied lesions showed complete response after calcium electroporation. Calcium electroporation is, based on the limited results of this study, currently not assessed as a suitable alternative treatment over surgical removal of sarcoids; however, calcium electroporation is deemed safe and feasible, and we observed a very good response rate in some of the sarcoids.

## Figures and Tables

**Table 1:** Overview of horses treated with calcium electroporation. Eight horses, six geldings and two mares, aged 2–15 years, were treated. The horses had between 1 and 9 sarcoids placed in the head and neck area, the extremities, and the ventral abdomen. The size of the sarcoids, the number of treatments, and the length of follow-up for each of the horses are shown.

No.	Sex	Age (Years)	No. of Sarcoids	Location of Sarcoids	Size of Sarcoids/Largest Diameter (min - max)	Number of Treatment(s) (Weeks Between Treatments)	Follow-up (After First Treatment)
1	gelding	11 years	3	Ventral abdomen, Extremities	2.4–3.5 cm	2 (13 weeks)	17 weeks
2	gelding	15 years	5	Head and neck, Ventral abdomen, Extremities	4.4–8.7 cm	1	Withdrawn from study due to multiple large mixed type sarcoids
3	gelding	4 years	3	Head and neck, Ventral abdomen	0.7–2.7 cm	2 (4 weeks)	25 weeks
4	gelding	8 years	1	Ventral abdomen	3.0 cm	1	38 weeks
5	gelding	3 years	9	Head and neck, Ventral abdomen, Extremities	0.9–5.0 cm	2 (4 weeks)	26 weeks
6	gelding	2 years	3	Ventral abdomen, Extremities	3.5–4.5 cm	2 (5 weeks)	22 weeks
7	mare	9 years	4	Head and neck	1.8–5.0 cm	2 (9 weeks)	32 weeks
8	mare	4 years	4	Ventral abdomen, Extremities	0.6–2.5 cm	2 (4 weeks)	12 weeks

**Table 2:** Overview of sarcoids treated with calcium electroporation. The median number of sarcoids per horse was 3.5, and they were situated mostly at the ventral abdomen followed by the extremities and head and neck area. The size of the sarcoids—both volume and largest diameter—is shown. The number of treatments, the injected volume of calcium, and the number of pulse series (8 pulses per series) are also shown. The types of sarcoids treated were fibroblastic and verrucous.

No. of Sarcoids Per Horse Median (min, max)	Placement of Sarcoids	Size of Sarcoids		Two Treatments	CaCl_2_ Injected/mL Mean (min, max)	No. of Pulse Series Mean (min, max)	Histology
		Volume/cm^3^ Mean (min, max)	Largest Diameter/cm Mean (min, max)				Type of Sarcoids
3.5 (1, 9)	Head and neck: 8 (25%) Ventral abdomen: 15 (47%) Extremities: 9 (28%)	6.3 cm^3^ (0.1, 54.5)	2.9 cm (0.6, 5.0)	6 of 8 horses 26 of 32 sarcoids	3.5 mL (0.5, 9.0)	19 (4, 53)	52% (11/21) fibroblastic 48% (10/21) verrucous
